# The aryl hydrocarbon receptor as a tumor modulator: mechanisms to therapy

**DOI:** 10.3389/fonc.2024.1375905

**Published:** 2024-05-14

**Authors:** Kanita A. Chaudhry, Anna Bianchi-Smiraglia

**Affiliations:** Department of Cell Stress Biology, Roswell Park Comprehensive Cancer Center, Buffalo, New York, NY, United States

**Keywords:** AhR, cancer biology, therapeutics, immune regulation, xenobiotic, tumor promoter, tumor suppressor

## Abstract

The aryl hydrocarbon receptor (AhR) is a ligand-activated transcription factor that is widely recognized to play important, but complex, modulatory roles in a variety of tumor types. In this review, we comprehensively summarize the increasingly controversial role of AhR as a tumor regulator and the mechanisms by which it alters tumor progression based on the cancer cell type. Finally, we discuss new and emerging strategies to therapeutically modulate AhR, focusing on novel agents that hold promise in current human clinical trials as well as existing FDA-approved drugs that could potentially be repurposed for cancer therapy.

## Introduction to AhR signaling

**Figure 1:**
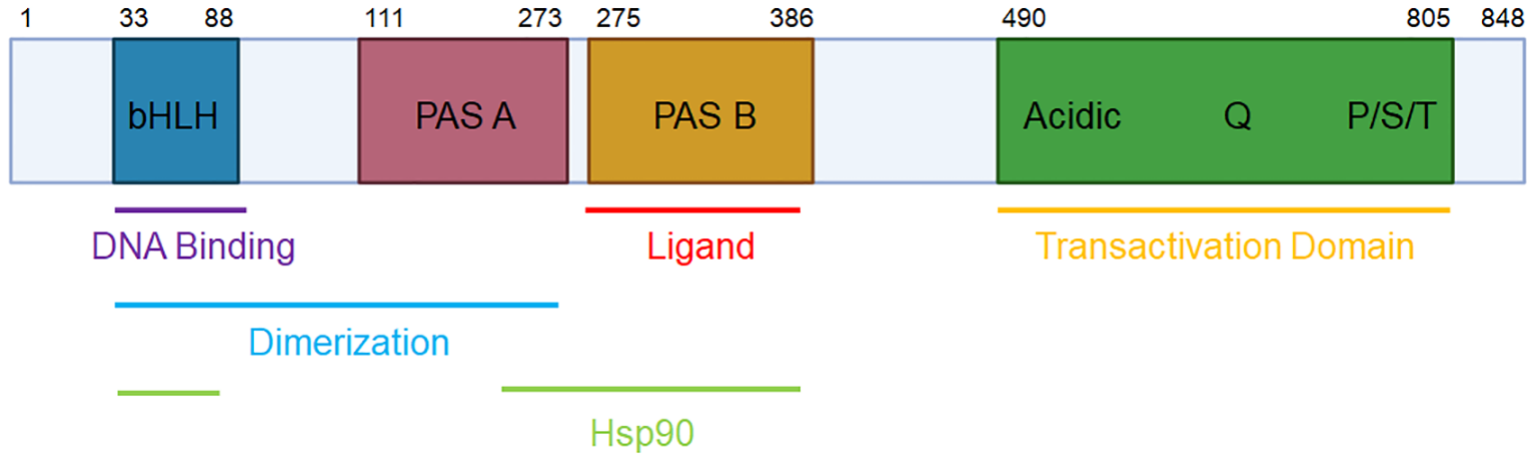
The domains of human AhR. The human AhR protein consists of an N-terminal basic helix-loop-helix (bHLH) domain for DNA binding, protein dimerization, and Hsp90 binding; a PAS-A domain for protein dimerization and Hsp90 binding; a PAS-B domain for ligand and Hsp90 binding; and a C-terminal transactivation domain.

The aryl hydrocarbon receptor (AhR) is a ligand-activated transcription factor that was identified in 1976 as the receptor mediating the effects of 2,3,7,8-tetrachlorodibenzo-*p*-dioxin (TCDD) (1). AhR is a member of the basic helix-loop-helix PER-ARNT-SIM (bHLH-PAS) family of transcription factors, and as such, coordinates transcriptional activity in response to environmental signals. The AhR protein is comprised of the N-terminal bHLH DNA-binding domain, two PAS domains for dimerization (with the PAS-B domain also responsible for ligand binding), and a C-terminal transactivation domain (2) (see **Figure 1**).

**Figure 2:**
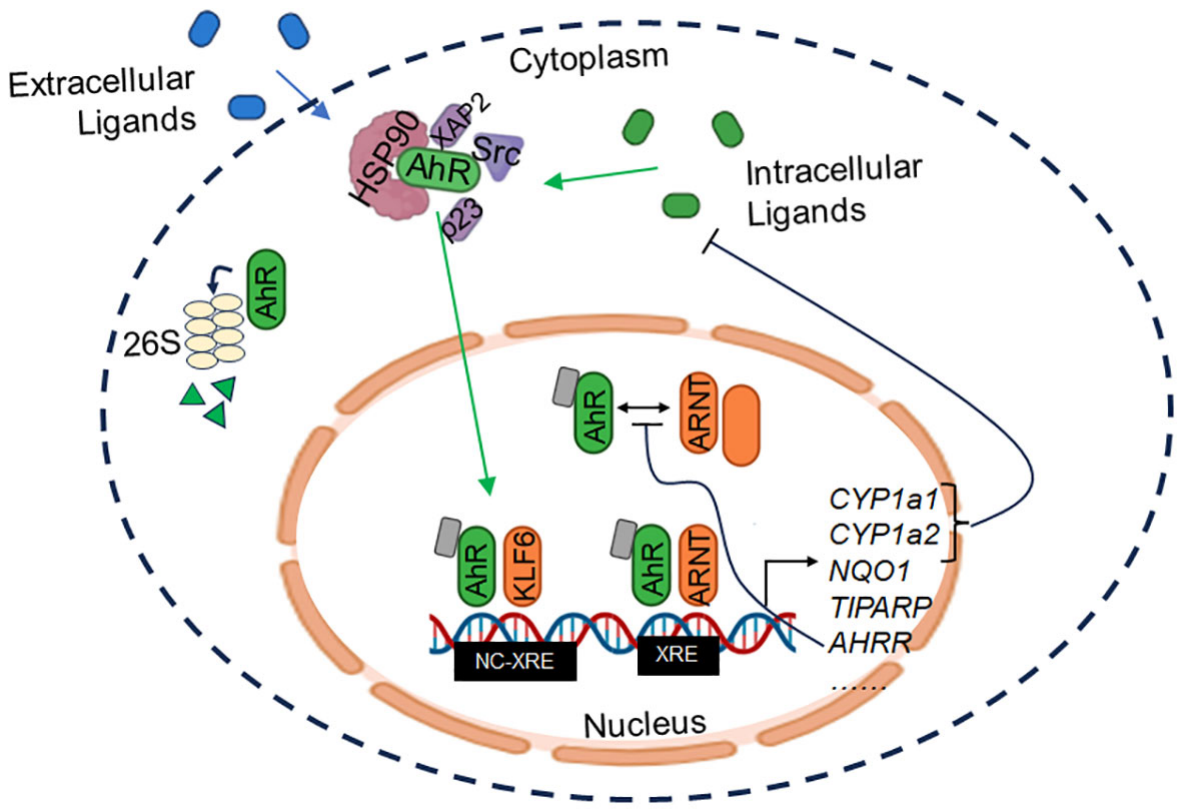
AhR signaling. AhR is normally sequestered in inactive form in the cytoplasm through interaction with chaperones (HSP90, XAP2, p23, and Src). Upon binding of exogenous or endogenous ligands, AhR dissociates from the complex and translocates to the nucleus where it dimerizes with ARNT or KLF6, among others, to induce transcription of target genes. XRE = xenobiotic response element; NC-XRE = non-canonical XRE.

AhR is normally sequestered in the cytoplasm where it is bound to chaperone proteins, notably heat shock protein 90 (Hsp90), X-associated protein 2 (XAP2), p23, and Src (3). Upon ligand binding, AhR translocates into the nucleus and forms a heterodimer with its canonical binding partner, AhR nuclear translocator (ARNT). The AhR-ARNT complex binds to xenobiotic response elements (XREs) within the DNA, leading to the induction of classical targets comprising the “AhR gene battery,” including cytochrome P450 enzymes *CYP1A1* and *CYP1A2*, ROS scavenger *NQO1*, poly(ADP-ribose) polymerase *TIPARP*, and the AhR repressor *AHRR*, among many others (4). AhR signaling is regulated via several mechanisms, including control of ligand availability by CYP enzymes (5). In a negative feedback loop, AhRR binds to ARNT, limiting AhR/ARNT transcriptional activity (6). Finally, AhR is degraded by the 26S proteasome, which is also triggered by AhR activation (7) (see **Figure 2**).

In recent years, it has become well-appreciated that AhR can bind a multitude of exogenous and endogenous ligands, heterodimerize with several non-canonical binding partners, and regulate diverse transcriptional programs. While xenobiotics such as TCDD (1) and polycyclic aromatic hydrocarbons (PAHs) (8) represent prototypical AhR agonists, an ever-growing list of exogenous and endogenous ligands have been described. In particular, tryptophan derivatives including 2-(1’H-indole-3’-carbonyl)-thiazole-4-carboxylic acid methyl ester (ITE) (9) as well as kynurenine (10) are among the most well-characterized endogenous AhR ligands. Accordingly, differences in ligand binding may differentially modulate AhR functionality (11). Indeed, AhR interacts with a diverse set of binding partners, such as RelA (12), estrogen receptor *α* (ER*α*) (13), Kruppel-like factor 6 (KLF6) (14), among others, at both xenobiotic response elements (XRE) and non-canonical XRE (nc-XRE) elements within the DNA, resulting in distinct gene expression changes.

*AHR*-deficient murine models have provided critical insight into AhR’s endogenous functions, revealing its role in the immune system, hepatic growth and development, and fertility (15). AhR-null mice remain viable and fertile, making them ideal models to study cancer development and progression.

### AhR in tumor biology

**Figure 3:**
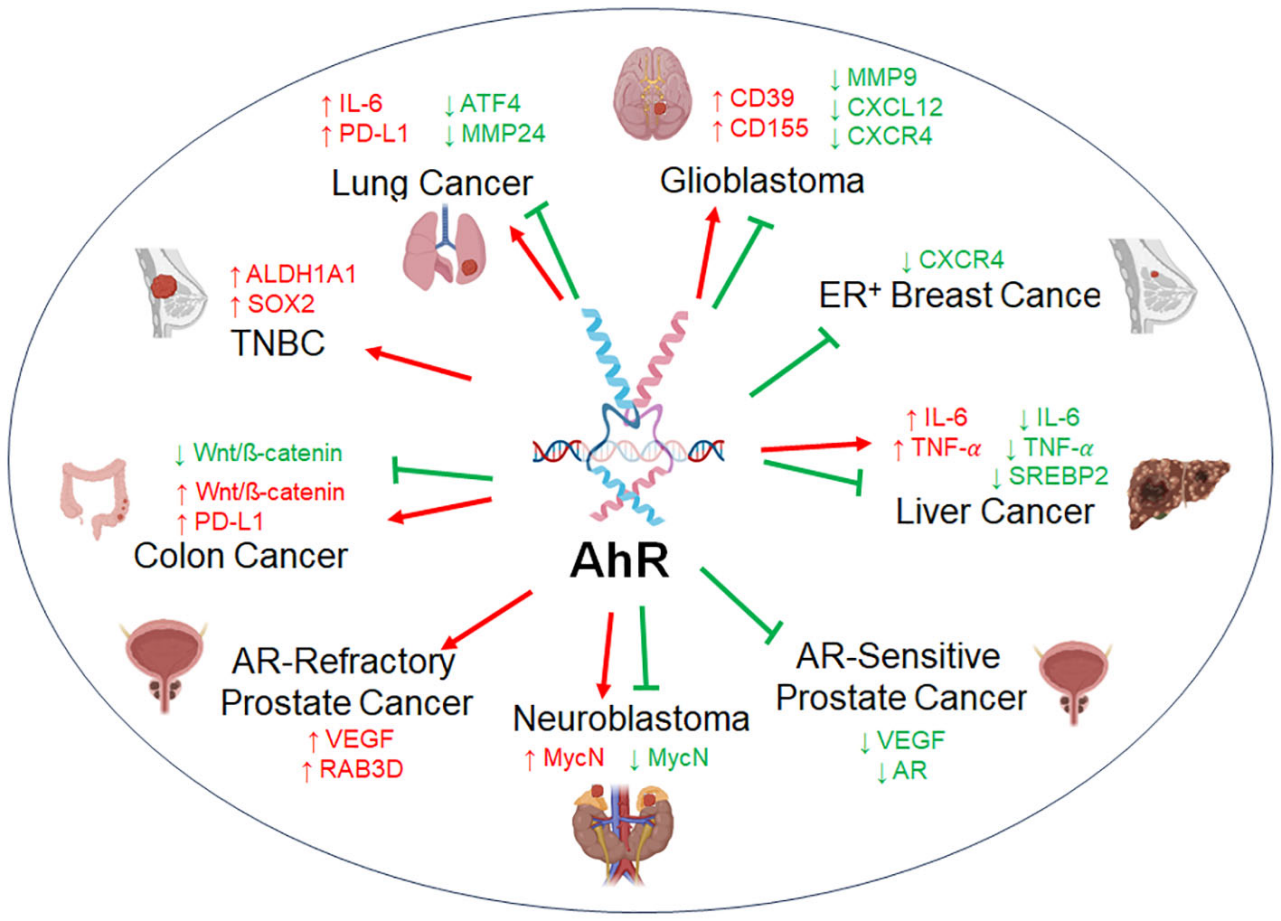
The dual role of AhR in tumor biology. AhR acts as a tumor promoter or as a tumor suppressor across representative cancer types. AhR regulates a variety of factors with either an oncogenic function (red) or anti-tumorigenic function (green), sometimes with opposing findings within the same subset of cancer.

AhR is ubiquitously expressed and dysregulated in a wide range of cancer types. Its function as a tumor modulator is complex, as AhR can act as pro-tumorigenic or anti-tumorigenic factor depending on the cancer cell type, sometimes, with conflicting reports (**Figure 3**). Here, we summarize the current state of knowledge of the tumor modulatory roles of AhR based on varying cancer subsets.

### Blood cancers

AhR is emerging as a tumor modulator in hematological malignancies, including acute myeloid leukemia (AML), multiple myeloma, chronic lymphocytic leukemia (CLL), and lymphomas, where its precise roles are just beginning to be understood. In AML, some reports suggest a tumor-promoting function of AhR, with high AhR expression and constitutive activity observed in AML patients (16). Scoville et al. (16) found that AML cells produce soluble ligands that activate AhR signaling in natural killer (NK) cells, resulting in the upregulation of miR-29b that impairs NK cell function. Consequently, AhR inhibition re-sensitizes AML cells to NK cell-induced cytotoxicity (16). On the contrary, other studies have shown reduced AhR signaling in AML cells, with AhR promoting AML differentiation, suppressing leukemic burden, and modulating AML resistance to bromodomain inhibitors (17). These discrepancies may be explained by differential ligands used among the studies; however, more work is needed to clarify how AhR alters AML disease progression.

Our group recently identified AhR as a poor prognostic factor in multiple myeloma patients that positively regulates the polyamine biosynthesis through transcriptional regulation of key players ornithine decarboxylase 1 (*ODC1*) and antizyme inhibitor 1 (*AZIN1*), and supports multiple myeloma cell proliferation (18). Subsequent corroborative studies by Hughes et al. (19) have demonstrated that AhR antagonism suppresses multiple myeloma cell viability, alters immune surface markers, and sensitizes multiple myeloma to NK cell cytotoxicity.

In CLL, microarray analysis revealed high *AHR* mRNA and target genes expression relative to other human B-cell lineage cancers, suggesting an oncogenic role for AhR in the disease (20). Consistently, CLL cells have been found to express indoleamine 2,3-dioxygenase 1 (*IDO1*) (21), which converts tryptophan to kynurenine, a ligand for AhR. IDO1-mediated kynurenine production rescues CLL cells from venetoclax-induced apoptosis and upregulates the pro-survival Mcl-1 in an AhR-dependent manner (21). Additionally, it was shown that the AhR-activating enzyme, IL4l1, is highly enriched in tumor-supportive monocytes in the Eµ-TCL1 mouse model of CLL and promotes CLL tumor progression (22). Thus, these findings imply that targeting AhR in the CLL tumor microenvironment could be an attractive therapeutic approach.

Studies in B and T-cell lymphomas have pointed to an oncogenic role for AhR. A recent report showed that AhR and its activating enzymes, IDO1 and tryptophan 2,3- dioxygenase (TDO), were highly expressed in diffuse large B-cell lymphoma (DLBCL) patient samples and were inversely correlated with patient survival (23). AhR localizes to the nucleus of DLBCL cell lines and drives expression of the germinal center oncogenes, *MEF2B* and *BCL6 (* 24). These studies raise the question of how endogenous ligands generated by IDO1 and TDO could modulate AhR activity in lymphomas.

### Breast cancer

Studies in breast cancer have revealed complex tumor regulatory functions for AhR. AhR is overexpressed and constitutively active in breast cancer (25), where it is thought to have varying effects depending on the ligand and cell type. It is well-established that there is an inhibitory crosstalk between AhR and estrogen receptor (ER) pathways, the mechanisms of which are described in great detail elsewhere (26) and also mentioned below. Consequently, ER status may influence the pro- or anti-tumorigenicity of AhR.

In triple-negative breast cancer – characterized by lack of expression of ER, progesterone receptor (PR), and lack of human epidermal growth factor receptor 2 (HER2) overexpression – TDO is particularly elevated and drives AhR activity to promote migration, anoikis resistance, and tumor metastasis (25, 27). Goode et al. (28) showed that shRNA-mediated depletion of AhR attenuates tumor growth in a xenograft human triple negative breast cancer (TNBC) MDA-MB-231 murine model. Stanford et al. (29) determined that AhR controls cancer stemness in human TNBC Hs578T cells, as AhR knockdown decreases tumor sphere formation and *in vivo* tumor growth via downregulation of *ALDH1A1* and *SOX2*. While these reports provide evidence that AhR plays an oncogenic role in triple-negative breast cancer, other studies describe opposite results. siRNA-mediated AhR knockdown has been found to increase MDA-MB-231 invasion (30). These discrepancies are further exacerbated by conflicting results obtained with AhR ligands that suggest AhR acts a tumor suppressor in triple-negative breast cancer. A wide array of AhR agonists including TCDD (31), 6-methyl-1,3,8-trichlorodibenzo-furan (MCDF) (31), and omeprazole (30) have repressed viability, proliferation, invasion, and/or metastasis in MDA-MB-231 or MDA-MB-468 cells in an AhR-dependent manner. These findings suggest that ligand-mediated activation of AhR in TNBC has differing effects than genetic AhR manipulation. This highlights the complexity of AhR functions in triple-negative breast cancer and necessitate further work to understand these differences.

In breast cancer subtypes where ER is positively expressed, AhR has been described to exert tumor suppressive functions. Köhle et al. found that expression of a constitutively active mutant of AhR impairs the estrogen-dependent growth of MCF-7 cells (32). Consistent with these data, numerous reports have shown that AhR ligands have anti-tumorigenic effects in ER^+^ breast cancer cells. For example, in MCF-7 cells, TCDD counteracts estrogen-mediated proliferation and G_1_/S phase cell cycle progression and suppresses xenograft tumor growth *in vivo (* 32, 33). Thus, there is consistent evidence from multiple laboratories that AhR is tumor suppressive in ER^+^ breast cancer cells. While this has generated efforts to therapeutically modulate AhR in ER^+^ patients, clinical trials have thus far not yielded success.

### Colon cancer

An increasing multitude of reports suggest a tumor regulatory role for AhR in colorectal cancer. A study by Kawajiri et al. (34) detected low AhR protein levels in human cecal cancer specimens, and determined that *AHR*-deficient mice harbor more cecal tumors than wild-type mice. Moreover, in the *APC^Min/+^* model of familial adenomatous polyposis, supplementation of the AhR ligands, indole-3-carbinol (I3C) and 3,3’-diindolylmethane (DIM), in the diet significantly delayed intestinal carcinogenesis (34). Other groups have corroborated a tumor suppressive function for AhR in colon cancer. Deletion of *AHR* in the *APC* ^S580/+^; *KRAS* ^G12D/+^ mouse model of colorectal cancer promotes proliferation and tumor growth and decreases mouse survival rate (35). At the same time, various other publications suggest a tumor promoting role for AhR in colon cancer. Recently, Miyazaki et al. (36) found that patient-derived colon cancer spheroids express high levels of *TDO2* and kynurenine. TDO2 promotes metastasis of colon cancer cells to the liver, upregulates programmed death ligand 1 (PD-L1) and suppresses immune responses, and maintains Wnt signaling in an AhR-dependent manner (36). Zhang et al. (37) showed that IDO1 expression in colon cancer cells stalls T cell proliferation. In a model of chronic colitis-associated cancer, *IDO1*-depleted mice have smaller and fewer tumors, reduced infiltrating regulatory T cells as well as increased CD8^+^ T cell abundance that is reversed with supplementation of kynurenine (37). Interestingly, gut microbiota such as *Fusobacterium nucleatum* produce formate, which drives metastatic dissemination, stemness, and increased Th17 cell infiltration via AhR signaling (38). Altogether, these findings reveal the complexity of AhR as a tumor modulator in colon cancer. While many *in vivo* studies suggest a tumor suppressive role, *in vitro* studies with ligands have generated opposing findings. This may be partly explained by the fact that tumor development in an *AHR*-deficient mouse model is distinct from AhR gain during tumorigenesis. Investigation into how the gut microbiome alters colorectal cancer progression is an emerging topic and further study will provide valuable insight into this research area.

### Esophageal and stomach cancers

In upper gastrointestinal tract tumors of the esophagus and stomach, a growing body of literature addresses the role for AhR in cancer progression. In esophageal cancer, various reports suggest AhR acts as a tumor promoter. It has been shown that AhR is highly expressed in patient-derived esophageal squamous cell cancers and correlates with poor overall survival (39). Genetic AhR overexpression promotes esophageal carcinoma migration and invasion via upregulation of phosphorylated epidermal growth factor receptor (*p*-EGFR) and RhoA/ROCK1 (39). Studies in mice demonstrate that esophageal squamous cell carcinoma cells express TDO, which promotes tumor growth and induces monocyte differentiation into the pro-tumorigenic M2 macrophage via AhR (40). While collectively these studies support a role for AhR in promoting esophageal carcinogenesis, differing observations were noted with ligand AhR activation. Treatment of esophageal squamous cell carcinoma cells with the AhR ligand 3,3’-diindolylmethane (DIM) represses proliferation, invasion, migration, and tumor growth in xenograft models (39).

A pro-tumorigenic role for AhR has also been largely reported in gastric cancers. Transgenic mice expressing constitutively active AhR develop hamartomatous tumors in the glandular part of the stomach, which is accompanied by a downregulation of osteopontin (41). AhR is strongly expressed and localized in the nucleus of human gastric cancer tissues and cell lines (42). Genetic depletion of AhR suppresses viability, proliferation, migration, and invasion of gastric cancer cells, and *in vivo* administration of the AhR inhibitor, biseugenol, prevents gastric tumor growth, metastasis, and peritoneal dissemination (42).

### Gynecologic malignancies

The role of AhR in gynecologic malignancies, including ovarian and uterine tumors, is just beginning to be understood. In human ovarian cancer tissues, immunohistochemistry analysis has shown positive AhR staining in a range of histological subtypes (43). IDO1 is also expressed by ovarian carcinomas where its levels are sustained by an autocrine AhR-IL-6-STAT3 signaling loop (44). *In vivo* experiments in mice have demonstrated that ovarian tumoral IDO1 mediates PD-1 upregulation on CD8^+^ T cells via AhR and causes infiltration of suppressive immune cells in the tumor microenvironment, augmenting ovarian tumor growth (45). These studies suggest a potential oncogenic role for AhR in ovarian cancer. However, treatment of ovarian cancer cell lines with AhR agonists appears to have varying effects depending on the ligand and cell type. For example, exposure of cells to the AhR agonist, ITE, decreases proliferation of OVCAR-3 cells and suppresses migration of SKOV-3 cells, but has no effect on the IOSE-385 cell line (43). As in breast cancer, these data may be partly explained by inhibitory crosstalk of the AhR signaling pathway with the estrogen receptor (ER) pathway (46). Further investigation is needed to clarify the tumorigenic role of AhR in ovarian cancers.

In endometrial cancer, mixed observations regarding the tumor regulatory role of AhR have been reported. Some studies suggest AhR plays an oncogenic role in endometrial cancer, as AhR is upregulated in human endometrial cancer lesions and its increased expression significantly correlates with higher tumor grade (47). Recent work by Li et al. (47) shows that genetic depletion of *AHR* reverts the growth, invasion, and motility induced by knockdown of the tumor suppressive transcription factor, nuclear factor 1-C (*NF1C*) in HHUA, HEC-6, and hEM cell lines, suggesting that NF1-C suppresses AhR-mediated tumorigenic functions. Ligand-induced AhR activation in endometrial cancer largely has anti-estrogenic and tumor suppressive effects, as seen in ER^+^ positive breast cancer. TCDD, B[a]P, and MCDF suppress estrogen-induced Ishikawa and ECC-1 cell proliferation. ITE reduces proliferation, migration, and *in vivo* tumor growth of AN3-CA, HCE-1B, and Ishikawa cells in an AhR-dependent manner (48). Again, these results highlight how AhR’s effects are strongly dependent on ligand and on cell type.

### Head and neck cancers

An increasingly important role for AhR as a tumor promoter has been demonstrated in various head and neck cancers. AhR is constitutively active in head and neck squamous carcinoma cells where it promotes migration and invasive capability, and drives expression of IL-6 and growth factors, including amphiregulin (AREG), epiregulin (EREG), and platelet-derived growth factor A (PDGFA) (49). Recently, Frank et al. (50) showed that antibiotic treatment of a chemically-induced mouse model of oral squamous cell carcinoma reduced AhR activity, raising the possibility that *Lactobacillus* spp., enriched in this cancer type, activates AhR (50). Similarly, exposure of oral squamous cell carcinoma cells to the supernatants from *Pseudomonas aeruginosa* and *Porphyromonas gingivalis*, the latter commonly found in the oral cavity, induced AhR activity and augmented expression of ALDH1, a marker associated with chemoresistance (51). Consequently, AhR antagonism increases sensitivity to cisplatin, decreases tumor sphere formation, and reduces xenograft tumor growth in oral squamous cell carcinoma (51). AhR also modulates the tumor microenvironment of oral squamous cell carcinoma. Kenison et al. (52) showed that AhR deletion in murine orthotopic oral cancer cells prevents tumor growth and decreases expression of inhibitory immune checkpoints PD-L1, CD39, CTLA-4, PD-1, and Lag3 on multiple immune cell types.

### Liver and pancreatic cancers

The tumor modulatory role of AhR is well-appreciated in liver cancers but remains controversial. Although most reports suggest a pro-tumorigenic function for AhR in hepatocellular carcinoma, some studies have documented a tumor suppressive role. It has been shown that AhR is highly expressed in human liver cancer tissues and cell lines (53). In the diethylnitrosamine (DEN)-induced mouse model of hepatic carcinogenesis, some groups found that constitutively active AhR expression promotes liver tumor formation (54). Conversely, another report showed that DEN-induced AhR-deficient mice have greater hepatic tumor incidence, increased proliferation, and higher IL-6 and TNF-*α* expression (55). Interestingly, there are also mixed findings regarding endogenous AhR activity in liver cancers. Some studies show the overexpression of TDO in human hepatocellular carcinoma patient tissues that drives cell growth, migration, invasion, and epithelial to mesenchymal transition (EMT) via the AhR pathway (56). However, a contrasting report found that TDO is downregulated in human hepatocellular carcinomas, inhibits cell proliferation, and represses tumor xenograft growth (57). Intriguingly, a recent study suggests that tryptophan metabolites produced by gut flora such as *Lactobacillus reuteri* attenuates expression of sterol regulatory element-binding protein 2 (SREBP2) and correspondingly prevents liver tumorigenesis in an AhR-dependent manner (58). In summary, these data illustrate that the tumor modulatory functions of AhR in liver cancers remain to be fully understood.

AhR has also been found to modulate pancreatic cancer progression. Early studies by Koliopanos et al. (59) demonstrated that AhR is overexpressed in human pancreatic cancer tissue specimens and cell lines. This work has shown that AhR activation by agonists – including TCDD, MCDF, and DIM – suppresses the growth of pancreatic cancer cells (59). However, more recent studies have suggested a tumor promoting role for AhR in pancreatic tumors. Human pancreatic cancers highly express IDO1 and TDO, which correlate with poor patient prognosis, and causes increased migration and invasion of cells and spheroids via kynurenine-mediated AhR activation (60). Consistently, metabolic profiling of pancreatic ductal adenocarcinoma patients revealed that higher kynurenine levels are associated with poorer overall survival of patients (61). Tumors expressing high levels of IDO1 are enriched for *AHR* pro-tumorigenic target genes, including *NFE2L2* (Nrf2), *SERPINB2*, *IL1b*, *IL6*, and *IL8 (* 61). Furthermore, AhR is expressed by tumor-associated macrophages where its activation by *Lactobacillus*-metabolized tryptophan metabolites drives immunosuppression and pancreatic tumor growth (62). Thus, while exogenous AhR activation appears to have a tumor suppressive effect, emerging evidence suggests that endogenous ligands may promote pancreatic cancer progression.

### Lung cancer

An increasingly visible role for AhR as a regulator of lung cancer tumorigenicity is being appreciated, with most reports focused on non-small cell lung cancers. A solid body of data published by various laboratories supports a pro-tumorigenic role for AhR in lung cancer. Nuclear AhR expression is significantly associated with poor survival of patients with non-small cell lung carcinoma (63). Consistently, AhR inhibition or knockdown sensitizes non-small cell lung cancer to EGFR tyrosine kinase inhibitors *in vitro* and *in vivo (* 63). Interestingly, Wang et al. (64) found that cigarette smoke upregulates PD-L1 via AhR in non-small cell lung cancer cells, and treatment with *α*-PD-L1 attenuates benzo-alpha-pyrene (BaP)-induced lung cancer *in vivo*. Accordingly, AhR inhibition with *α*-naphthoflavone (ANF) significantly enhances the efficacy of *α*-PD-L1 in lung cancer mouse models and prolongs the lifespan of mice (64). While these studies provide evidence that AhR acts as a tumor promoter, there are also reports suggesting a tumor suppressive function for AhR in lung cancer. Nothdurft et al. (65) determined that *AHR* depletion by shRNA augments invasiveness *in vitro* and metastatic capability of non-small cell lung cancer cells *in vivo* via regulation of the EMT pathway, ATF4 signaling, and MMP24 expression. AhR has also been shown to inhibit tumor growth and suppress the expansion of lung progenitor cells in a murine model of KRAS*^G12D^*-induced non-small cell lung cancer (66). Thus, the role of AhR in lung cancer requires further study to fully understand its tumorigenic functions.

### Nervous system tumors

AhR has been reported to play increasingly important roles in central and peripheral nervous system tumors. In the context of malignant gliomas, including glioblastoma, some groups have described AhR as a tumor promoter (10, 22, 67, 68), while others have shown that AhR acts as a tumor suppressor (69). Opitz et al. (10) originally established TDO-derived kynurenine produced by human gliomas as an oncometabolite that activates AhR, supports glioma cell survival and motility, and suppresses immune responses. AhR drives CD39 and CD155 expression in tumor-associated macrophages, thereby impairing T cell response in glioblastoma (68). Interestingly, *R*-2-hydroxyglutarate (*R*-2-HG), a metabolite that accumulates in isocitrate dehydrogenase (IDH)-mutant gliomas, enzymatically induces TDO in tumor-infiltrating myeloid cells, leading to AhR-dependent suppression of macrophage function and anti-tumor immunity (67). On the other hand, conflicting reports have demonstrated that kynurenine does not alter AhR activity or invasion of glioblastoma cells, and that the AhR antagonist, CH-223191, inhibits glioblastoma invasion in an AhR-independent manner in both AhR-expressing and AhR-silent cell lines (69). These studies show that AhR knockdown increases glioblastoma cell invasion and migration by induction of MMP9, CXCL12, and CXCR4 (69). Collectively, while these studies demonstrate the importance of AhR in malignant gliomas, more research is needed to understand its tumor modulatory roles that may shed light into contradictory data reported by different laboratories.

The role of AhR in neuroblastoma is largely understudied. Recent work from our laboratory suggests AhR acts as a tumor promoter in *MYCN*-amplified neuroblastoma (70). We have found that AhR transcriptional activity correlates with poor patient prognosis, positively regulates MycN, and represses differentiation of *MYCN*-amplified neuroblastoma cells by altering chromatin accessibility and modulating the retinoic acid receptor pathway (70). Accordingly, AhR antagonism with the AhR antagonist clofazimine (CLF) synergistically augments retinoic acid-induced differentiation (70), suggesting that modulation of AhR may be a potential and promising therapeutic approach for improving standard-of-care in this disease. We and others have also reported that AhR has a tumor suppressive role in non-*MYCN*-amplified neuroblastoma cells. Treatment of non-*MYCN*-amplified neuroblastoma cells with TCDD has been found to induce non-apoptotic cell death via an AhR-dependent mechanism (71). Wu et al. reported that AhR induces cell differentiation, reduces *in vivo* tumor burden, and downregulates MycN expression in the context of non-*MYCN*-amplified neuroblastoma (72, 73). Our work revealed that AhR suppresses cMYC in similar systems (70). Thus, current evidence lends support to the hypothesis that AhR has a dual tumor modulatory role depending on the neuroblastoma subtype.

### Prostate cancer

In prostate cancer, the tumor modulatory effects of AhR appear to be context-dependent, differing based on androgen sensitivity or refractoriness. Multiple lines of evidence suggest AhR exerts tumor suppressive effects in androgen-sensitive prostate cancer, with some mixed reports. Studies in the transgenic, androgen-sensitive TRAMP model of prostate cancer show that AhR protects against prostate cancer development, as *AHR* ^+/+^ TRAMP mice exhibit lower tumor formation than *AHR* ^-/-^ or *AHR* ^+/-^ TRAMP mice (74). In line with these findings, treatment of TRAMP mice with the selective AhR modulator, 6-methyl-1,3,8-trichlorodibenzo-furan (6-MCDF), which displays AhR agonist activity in prostate cell lines, inhibits prostate metastatic ability (75). *In vitro* experiments with AhR agonists in androgen-sensitive human prostate cancer cells have corroborated these murine studies. The AhR agonist, carbidopa, suppresses LNCaP proliferation and induces AhR-mediated proteasomal degradation of androgen receptor (AR) (76).

In androgen-refractory prostate cancer models, however, AhR has been suggested to act as a tumor promoter. AhR is highly expressed and constitutively active in androgen-independent (hormone-refractory) prostate cancer cells relative to androgen-sensitive prostate cancer cells (77). Immunohistochemistry of human prostate cancer tissues shows increasing AhR expression with tumor grade (77). AhR depletion or inhibition decreases cell proliferation, reduces AR protein levels, and inhibits AR target gene expression in androgen-independent cells (77). Further studies are needed to understand the crosstalk of the AhR and AR pathways and how they impact prostate cancer progression in both androgen-sensitive and androgen-refractory settings.

### Skin cancers

Several studies imply a tumor regulatory role for AhR in melanoma. Genome-wide association studies have suggested that the *AHR* gene locus at 7p.21.1 confers susceptibility to cutaneous malignant melanoma (78). Recent work has shown that AhR drives resistance to BRAF inhibitors in melanoma, and that AhR inhibition with resveratrol and flavinoids re-sensitize melanoma to BRAF inhibition (79). Although these studies demonstrate an oncogenic role for AhR, there are also some reports indicating that AhR has a tumor suppressive role in melanoma. In particular, Contador-Troca et al. (80) found that AhR suppresses growth and metastasis of melanoma *in vivo*. Immunohistochemistry revealed that human melanoma patient samples express lower levels of AhR compared to human nevi (80). Interestingly, Bender et al. found that tryptophan-derived metabolites derived from *Lactobacillus reuteri* promote immune-checkpoint inhibitor efficacy in melanoma via activation of the AhR (81).

### Urothelial tumors

Recent reports suggest a pro-tumorigenic role for AhR in urothelial cancers. Through analysis of whole-genome sequencing data, Vlaar et al. (82) identified recurrent in-frame deletions in exons 8 and 9 of *AHR* (*AHR* ^Δe8-9^) in 10% of metastatic urothelial cancer patients. *AHR* ^Δe8-9^ causes ligand-independent AhR activation and anchorage-independent growth of bladder organoids, acting as a novel driver mutation (82). In addition, Shi et al. (83) newly characterized *AHR* ^Q383H^ as an apolipoprotein B mRNA-editing enzyme catalytic polypeptide-like (APOBEC)-associated driver hotspot mutation in bladder cancer. *AHR* ^Q383H^ leads to higher sensitivity of AhR to ligand activation, greater AhR activity, and increased dependency of luminal bladder cancer cells on AhR for cell survival (82, 83). High expression of AhR and its target genes, *CYP1A1* and *CYP1A2*, has been correlated with increased histological grade, tumor stage, and progression in muscle-invasive bladder cancer and upper urinary tract cancers (84).

### AhR and immunity

Notably, AhR not only contributes to tumor growth and survival in a tumor-intrinsic fashion as highlighted in the chapters above, but multiple recent publications have revealed an AhR-dependent regulation of the tumor microenvironment, especially with regard to the immune system, to modulate tumor immune surveillance and allow escape from immunosuppression. A plethora of endogenous and exogenous AhR ligands can be found in the tumor microenvironment due to tumor cells’ related metabolism, the organismal microbiota metabolism, as well as dietary consumption and pollutants absorption. In most cases, AhR activation results in the alteration of the tumor-immune cell interactions, with suppression of anti-tumor functions and induction of tumor-permissive or tumor-promoting immune landscapes, as AhR has been shown to regulate the differentiation of multiple cell types in both the innate and the adaptive immune response compartments. Several recent reviews have extensively described the role of AhR in the regulation of the immune system during cancer progression (85–88); thus, here we will briefly summarize some of the recent key findings.

AhR activation has been found to promote the trans-differentiation of antigen-presenting cells (APCs), such as dendritic cells (DCs) and macrophages, toward a more tolerogenic or tumor-permissive phenotype, resulting in the generation of immune-suppressive regulatory T (Tregs) cells. Hezaveh et al. reported that the activation of AhR in tumor-associated macrophages (TAMs) from microbiome-produced tryptophan metabolites leads to immunosuppression in pancreatic ductal adenocarcinoma (PDAC) thus promoting tumor growth (62). In the same study, it was found that, conversely, AhR pharmacological antagonism, as well as AhR deletion from myeloid cells, results in improved T-cell tumor infiltration, leading to tumor suppression (62). Likewise, Sadik et al. revealed that interleukin 4-induced 1 (IL4I1) can generate AhR ligands such as indole metabolites and kynurenic acid, which result in the suppression of adaptive immunity (22). Interestingly, immune checkpoint inhibitors (ICIs) whose use has been incorporated in the standard-of-care of multiple cancer types, were found to induce IL4I1 and activate AhR, thus generating a negative feedback loop (22). Inadvertent AhR activation by chemotherapy, resulting in suppression of innate immunity responses (i.e interferon type I production) has been recently reported also in triple negative breast cancer (89).

Work by Wu et al. (90) revealed the presence of an ARID5A-IDO1-AhR axis that leads to chimeric antigen receptor T-cell (CAR-T) exhaustion and immune evasion in colorectal cancer. Additionally, the authors performed a pan-cancer analysis which revealed high AhR expression in tumor-infiltrating immune cells, where AhR antagonism with a novel compound (BAY-2416964 (91)), see section below) showed promising potential in restoring immune cell function and enhancing antigen-specific cytotoxic T cell responses (90).

The role that AhR plays in the development and cytotoxic capabilities of natural killer (NK) cells is still complex and controversial, with some groups proposing AhR activation to be critical for proper NK cells cytotoxicity and functions (92, 93) and others claiming that AhR activation dampens NK cells activity instead (94).

While more work needs to be done to address the discrepancies, the consensus seems to be that AhR antagonism would be beneficial to promote a tumor-suppressive microenvironment and relieve the immune-suppression often seen associated with the more aggressive tumors.

## Therapeutic modulation of AhR in cancer

Given the well-recognized role of AhR as a regulator of tumor biology, many efforts are focused on developing therapeutic strategies to modulate AhR in cancer patients. Notably, this includes synthesizing novel AhR modulators as well as repurposing existing agents for the treatment of cancer patients. Certain agents, including the AhR agonist aminoflavone (AFP464) and the AhR antagonist StemRegenin-1 (SR1) (95), have been tested in clinical trials for breast neoplasms and solid tumors (NCT01015521, NCT00369200, NCT01233947) or hematological malignancies (NCT02765997), respectively. However, these trials were either withdrawn or terminated due to toxicity and poor side effect profiles. Currently, phase 1 clinical trials are ongoing for a novel AhR antagonist, BAY-2416964 (96), developed by Bayer and researchers at the German Cancer Research Center (DKFZ) for patients with advanced solid tumors (NCT04069026, NCT04999202). Preliminary reports presented at ASCO 2023 showed that BAY-2416964 seems to be well tolerated, suppresses AhR activation *in vivo*, and modulates immune functions. The authors noted that of 67 patients evaluable for response by RECIST, 22 (32.8%) had stable disease (91). Additionally, Ikena Oncology recently developed the novel AhR inhibitor, IK-175 (97), which is in phase 1 trials for urothelial cancers and solid tumors (NCT04200963 and NCT05472506) and received FDA Fast Track Designation. While not yet approved for cancer therapy, the AhR agonist tapinarof (98) is in clinical trials for atopic dermatitis and plaque psoriasis (NCT05142774, NCT05680740) and could potentially be tested for the treatment of dermatological malignancies in the future. These up-and-coming trials are promising and could represent the first AhR modulators to be clinically approved in patients for cancer therapy.

There are a growing number of FDA-approved drugs approved for other indications that have been recognized as AhR modulators, with demonstrated anti-tumor efficacy in pre-clinical studies (see **Table 1**). These include carbidopa (DOPA decarboxylase inhibitor) (76, 99, 100), dopamine (adrenergic receptor agonist) (101), flutamide (anti-androgen) (102), leflunomide (anti-rheumatic agent) (103, 104), nimodipine (anti-hypertensive agent) (30, 104), omeprazole (proton pump inhibitor) (30, 105, 106), raloxifene (estrogen modulator) (108), sorafenib (kinase inhibitor) (109), sulindac (nonsteroid anti-inflammatory) (30, 104, 110), transilast (anti-allergic agent) (107), and vemurafenib (BRAF inhibitor) (79). Our group previously identified clofazimine (CLF), an FDA-approved antibiotic approved for drug-resistant tuberculosis and lepromatous leprosy, as a novel AhR antagonist with anti-multiple myeloma and anti-neuroblastoma efficacy (18, 70). Importantly, the favorable safety profiles of these already FDA-approved agents warrant their testing in clinical trials for cancer patients, as they hold promise as anti-cancer drugs that could be rapidly translated into the clinic.

**Table 1:** Clinically approved AhR modulators for cancer therapy.

Agent	Mode of AhR Modulation	Cancer Type(s)	Reference(s)	Clinical Trial	FDA Approval
BAY-2416964 (96)	Antagonist	Head and neck, Lung, Colon	96	NCT04069026	No
Carbidopa (76, 99, 100)	Agonist	Pancreatic, Breast, Prostate	76, 99, 100		
Clofazimine (18, 70)	Antagonist	Multiple myeloma, Neuroblastoma	18, 70		Yes
Dopamine (101)	Agonist	Glioblastoma, Colon, Pancreatic	101		Yes
Flutamide (102)	Agonist	Liver cancer	102		Yes
IK-175 (97)	Inhibitor	Urothelial	97	NCT04200963	No
Leflunomide (103, 104)	Agonist/Partial Agonist	Melanoma, Breast	103, 104		
Nimodipine (30, 104)	Agonist	Breast	30, 104		Yes
Omeprazole (30, 105–107)	Agonist	Pancreatic, Breast, Esophageal, Glioblastoma	30, 105–107		Yes
Raloxifene (108)	Agonist	Breast, Liver	108		Yes
Sorafenib (109)	Antagonist	Liver, Ovarian	109		Yes
Sulindac (30, 104, 110)	Agonist	Breast, Colon	30, 104, 110		Yes
StemRegenin-1 (95)	Antagonist	Acute myeloid leukemia, acute lymphocytic leukemia, chronic myelogenous leukemia	95		No
Tapinarof (98)	Agonist	Atopic Dermatitis, Plaque Psoriasis	98	NCT05680740, NCT05142774	Yes
Transilast (107)	Agonist/Partial Agonist	Breast, Pancreatic	107		No
Vemurafenib (79)	Agonist/ Antagonist	Breast, Melanoma	79		Yes

It is important to note, however, that AhR antagonism may not benefit all type of cancers as, as noted in the sections above, in a subset of diseases AhR has been shown to act as a tumor suppressor (i.e. ER+ breast cancer, androgen-sensitive prostate cancer, non-*MYCN*-amplified neuroblastoma, see **Figure 3**). Additionally, while not many severe side effects have been revealed so far by the use of AhR antagonists in current clinical trials, systemic inhibition of AhR may still end up causing co-morbidities later in life, as AhR has well-documented physiological roles in normal development, as well as still controversial roles in some of the immune cell populations (i.e. NK cells).

## Conclusions and future perspectives

A tremendous body of literature continues to provide support for AhR as a critical modulator of tumor progression across a wide variety of cancer types – from solid and liquid tumors to adult and pediatric malignancies. Like many other transcription factors, AhR has a dual role in cancer biology, with either oncogenic or tumor suppressive effects that are highly contextual, depending on the specific ligand or cell type. While these context-specific effects have often produced conflicting results, they also provide avenues for further investigation. The ligand-specific effects of endogenous ligands, such as those produced by gut microbiota, on tumor growth is an exciting and emerging topic with potential therapeutic applications. Finally, the development and clinical testing of novel AhR modulating drugs, such as the FDA Fast Track-Designated AhR inhibitor, IK-175, are promising and could represent potential new cancer therapies.

## Author contributions

AB-S: Writing – original draft, Writing – review & editing. KC: Writing – original draft, Writing – review & editing.

## Glossary

**Table:**

AhR	aryl hydrocarbon receptor
TCDD	2,3,7,8-tetrachlorodibenzo-p-dioxin
bHLH	basic helix-loop-helix
HSP90	heath shock protein 90
XAP2	X-associated protein 2
ARNT	AhR nuclear translocator
AHRR	AhR repressor
PAH	polycyclic aromatic hydrocarbons
ITE	2-(1’H-indole-3’-carbonyl)-thiazole-4-carboxylic acid methyl ester
ER	estrogen receptor
PR	progesterone receptor
HER2	human epidermal growth factor receptor 2
KLF6	Kruppel-like factor 6
AML	acute myeloid leukemia
CLL	chronic lymphocytic leukemia
NK	natural killer cells
ODC1	ornithine decarboxylase 1
AZIN1	antizyme inhibitor 1
IDO1	indoleamine 2,3-dioxygenase 1
TDO	tryptophan 2,3- dioxygenase
DLBCL	diffuse large B-cell lymphoma
TNBC	triple negative breast cancer
shRNA	short-hairpin RNA
siRNA	small-inhibiting RNA
MCDF	6-methyl-1,3,8-trichlorodibenzo-furan
I3C	indole-3-carbinol
DIM	3,3’-diindolylmethane
PD-1	programmed death receptor 1
PD-L1	programmed death ligand 1
EGFR	epidermal growth factor receptor
NF1C	nuclear factor 1-C
AREG	amphiregulin
EREG	epiregulin
PDGFA	platelet-derived growth factor A
DEN	diethylnitrosamine
EMT	epithelial-to-mesenchymal transition
SREBP2	sterol regulatory element-binding protein 2
BaP	benzo-alpha-pyrene
R-2-HG	R-2-hydroxyglutarate
IDH	isocitrate dehydrogenase
CLF	clofazimine
6-MCDF	6-methyl-1,3,8-trichlorodibenzo-furan
AR	androgen receptor
Tregs	regulatory T cells
CAR-T	chimeric antigen receptor T cells.

## References

[1] Poland A Glover E Kende AS. Stereospecific, high affinity binding of 2,3,7,8 tetrachlorodibenzo p dioxin by hepatic cytosol. Evidence that the binding species is receptor for induction of aryl hydrocarbon hydroxylase. J Biol Chem. (1976) 251:4936–46. doi: 10.1016/s0021-9258(17)33205-2 956169

[2] Fukunaga BN Probst MR Reisz-Porszasz S Hankinson O. Identification of functional domains of the aryl hydrocarbon receptor. J Biol Chem. (1995) 270:29270–8. doi: 10.1074/jbc.270.49.29270 7493958

[3] Gruszczyk J Grandvuillemin L Lai-Kee-Him J Paloni M Savva CG Germain P. Cryo-EM structure of the agonist-bound Hsp90-XAP2-AHR cytosolic complex. Nat Commun. (2022) 13:1–13. doi: 10.2210/pdb7zub/pdb PMC9668932 36385050

[4] Nebert DW Roe AL Zieter MZ Solis WA Yang Y Dalton TP. Role of the aromatic hydrocarbon receptor and [Ah] gene battery in the oxidative stress response, cell cycle control, and apoptosis. Biochem Pharmacol. (2000) 59:66–85. doi: 10.1016/S0006-2952(99)00310-X 10605936

[5] Schiering C Wincent E Metidji A Iseppon A Li Y Alexandre J. Feedback control of AHR signaling regulates intestinal immunity. Nature. (2017) 542:242–5. doi: 10.1038/nature21080.Feedback PMC5302159 28146477

[6] Mimura J Ema M Sogawa K Fujii-Kuriyama Y. Identification of a novel mechanism of regulation of Ah (dioxin) receptor function. Genes Dev. (1999) 13:20–5. doi: 10.1101/gad.13.1.20 PMC316371 9887096

[7] Davarinos NA Pollenz RS. Aryl hydrocarbon receptor imported into the nucleus following ligand binding is rapidly degraded via the cytosplasmic proteasome following nuclear export. J Biol Chem. (1999) 274:28708–15. doi: 10.1074/jbc.274.40.28708 10497241

[8] Shimada T Inoue K Suzuki Y Kawai T Azuma E Nakajima T. Arylhydrocarbon receptor-dependent induction of liver and lung cytochromes P450 1A1, 1A2, and 1B1 by polycyclic aromatic hydrocarbons and polychlorinated biphenyls in genetically engineered C57BL/6J mice. Carcinogenesis. (2002) 23:1199–207. doi: 10.1093/carcin/23.7.1199 12117779

[9] Song J Clagett-Dame M Peterson RE Hahn ME Westler WM Sicinski RR. A ligand for the aryl hydrocarbon receptor isolated from lung. Proc Natl Acad Sci U S A. (2002) 99:14694–9. doi: 10.1073/pnas.232562899 PMC137481 12409613

[10] Opitz CA Litzenburger UM Sahm F Ott M Tritschler I Trump S. An endogenous tumour-promoting ligand of the human aryl hydrocarbon receptor. Nature. (2011) 478:197–203. doi: 10.1038/nature10491 21976023

[11] Denison MS Soshilov AA He G Degroot DE Zhao B. Exactly the same but different: Promiscuity and diversity in the molecular mechanisms of action of the aryl hydrocarbon (dioxin) receptor. Toxicol Sci. (2011) 124:1–22. doi: 10.1093/toxsci/kfr218 21908767 PMC3196658

[12] Kim DW Gazourian L Quadri SA Raphaëlle Sherr DH Sonenshein GE. The RelA NF-κB subunit and the aryl hydrocarbon receptor (AhR) cooperate to transactivate the c-myc promoter in mammary cells. Oncogene. (2000) 19:5498–506. doi: 10.1038/sj.onc.1203945 11114727

[13] Beischlag TV Perdew GH. ERα-AHR-ARNT protein-protein interactions mediate estradiol-dependent transrepression of dioxin-inducible gene transcription. J Biol Chem. (2005) 280:21607–11. doi: 10.1074/jbc.C500090200 15837795

[14] Wilson SR Joshi AD Elferink CJ. The tumor suppressor kruppel-like factor 6 is a novel aryl hydrocarbon receptor DNA binding partner. J Pharmacol Exp Ther. (2013) 345:419–29. doi: 10.1124/jpet.113.203786 PMC3657114 23512538

[15] Schmidt JV Su GHT Reddy JK Simon MC Bradfield CA. Characterization of a murine Ahr null allele: Involvement of the Ah receptor in hepatic growth and development. Proc Natl Acad Sci U S A. (1996) 93:6731–6. doi: 10.1073/pnas.93.13.6731 PMC39095 8692887

[16] Scoville SD Nalin AP Chen L Chen L Zhang MH McConnell K. Human AML activates the aryl hydrocarbon receptor pathway to impair NK cell development and function. Blood. (2018) 132:1792–804. doi: 10.1182/blood-2018-03-838474 PMC6202909 30158248

[17] Romine KA Nechiporuk T Bottomly D Jeng S McWeeney SK Kaempf A. Monocytic differentiation and AHR signaling as primary nodes of BET inhibitor response in acute myeloid leukemia. Blood Cancer Discovery. (2021) 2:518–31. doi: 10.1158/2643-3230.bcd-21-0012 PMC8462123 34568834

[18] Bianchi-Smiraglia A Bagati A Fink EE Affronti HC Lipchick BC Moparthy S. Inhibition of the aryl hydrocarbon receptor/polyamine biosynthesis axis suppresses multiple myeloma. J Clin Invest. (2018) 128:4682–96. doi: 10.1172/JCI70712 PMC6159960 30198908

[19] Hughes T Cottini F Catton E Ciarlariello D Chen L Yang Y. Functional expression of aryl hydrocarbon receptor as a potential novel therapeutic target in human multiple myeloma. Leuk Lymphoma. (2021) 62:2968–80. doi: 10.1080/10428194.2021.1948033 34232800

[20] Sherr DH Monti S. The role of the aryl hydrocarbon receptor in normal and Malignant B cell development. Semin Immunopathol. (2013) 35:705–16. doi: 10.1007/s00281-013-0390-8 PMC3824572 23942720

[21] Atene CG Fiorcari S Mesini N Alboni S Martinelli S Maccaferri M. Indoleamine 2, 3-dioxygenase 1 mediates survival signals in chronic lymphocytic leukemia via kynurenine/aryl hydrocarbon receptor-mediated MCL1 modulation. Front Immunol. (2022) 13:832263. doi: 10.3389/fimmu.2022.832263 35371054 PMC8971515

[22] Sadik A Somarribas Patterson LF Öztürk S Mohapatra SR Panitz V Secker PF. IL4I1 is a metabolic immune checkpoint that activates the AHR and promotes tumor progression. Cell. (2020) 182:1252–1270.e34. doi: 10.1016/j.cell.2020.07.038 32818467

[23] Chen X Zang Y Li D Guo J Wang Y Lin Y. IDO, TDO, and AHR overexpression is associated with poor outcome in diffuse large B-cell lymphoma patients in the rituximab era. Med (United States). (2020) 99:E19883. doi: 10.1097/MD.0000000000019883 PMC7249864 32481253

[24] Ding J Dirks WG Ehrentraut S Geffers R MacLeod RAF Nagel S. BCL6 – Regulated by AhR/ARNT and wild-type MEF2B – Drives expression of germinal center markers MYBL1 and LMO2. Haematologica. (2015) 100:801–9. doi: 10.3324/haematol.2014.120048 PMC4450626 25769544

[25] Novikov O Wang Z Stanford EA Parks AJ Ramirez-Cardenas A Landesman. An aryl hydrocarbon receptor-mediated amplification loop that enforces cell migration in ER-/PR-/Her2- human breast cancer cells. Mol Pharmacol. (2016) 90:674–88. doi: 10.1124/mol.116.105361 PMC5074452 27573671

[26] Tarnow P Tralau T Luch A. Chemical activation of estrogen and aryl hydrocarbon receptor signaling pathways and their interaction in toxicology and metabolism. Expert Opin Drug Metab Toxicol. (2019) 15:219–29. doi: 10.1080/17425255.2019.1569627 30644759

[27] D’Amato NC Rogers TJ Gordon MA Greene LI Cochrane DR Spoelstra NS. A TDO2-AhR signaling axis facilitates anoikis resistance and metastasis in triple-negative breast cancer. Cancer Res. (2015) 75:4651–64. doi: 10.1158/0008-5472.CAN-15-2011 PMC4631670 26363006

[28] Goode GD Ballard BR Manning HC Freeman ML Kang Y Eltom SE. Knockdown of aberrantly upregulated aryl hydrocarbon receptor reduces tumor growth and metastasis of MDA-MB-231 human breast cancer cell line. Int J Cancer. (2013) 133:2769–80. doi: 10.1002/ijc.28297 PMC3797219 23733406

[29] Stanford EA Wang Z Novikov O Mulas F Landesman-Bollag E Monti S. The role of the aryl hydrocarbon receptor in the development of cells with the molecular and functional characteristics of cancer stem-like cells. BMC Biol. (2016) 14:1–22. doi: 10.1186/s12915-016-0240-y 26984638 PMC4794823

[30] Jin UH Lee SO Pfent C Safe S. The aryl hydrocarbon receptor ligand omeprazole inhibits breast cancer cell invasion and metastasis. BMC Cancer. (2014) 14. doi: 10.1186/1471-2407-14-498 PMC4226953 25011475

[31] Zhang S Kim KH Jin UH Pfent C Cao H Amendt B. Aryl hydrocarbon receptor agonists induce microRNA-335 expression and inhibit lung metastasis of estrogen receptor negative breast cancer cells. Mol Cancer Ther. (2012) 11:108–18. doi: 10.1158/1535-7163.MCT-11-0548 PMC3256275 22034498

[32] Köhle C Hassepass I Bock-Hennig BS Walter Bock K Poellinger L McGuire J. Conditional expression of a constitutively active aryl hydrocarbon receptor in MCF-7 human breast cancer cells. Arch Biochem Biophys. (2002) 402:172–9. doi: 10.1016/S0003-9861(02)00076-0 12051661

[33] Gierthy JF Bennett JA Bradley LM Cutler DS. Correlation of in vitro and in vivo growth suppression of MCF-7 human breast cancer by 2,3,7,8-tetrachlorodibenzo-p-dioxin. Cancer Res. (1993) 53:3149–53. 8319224

[34] Kawajiri K Kobayashi Y Ohtake F Ikuta T Matsushima Y Mimura J. Aryl hydrocarbon receptor suppresses intestinal carcinogenesis in Apc Min/+ mice with natural ligands. Proc Natl Acad Sci U S A. (2009) 106:13481–6. doi: 10.1073/pnas.0902132106 PMC2726415 19651607

[35] Han H Davidson LA Hensel M Yoo G Landrock K Allred C. Loss of aryl hydrocarbon receptor promotes colon tumorigenesis in ApcS580/þ; KrasG12D/þ mice. Mol Cancer Res. (2021) 19:771–83. doi: 10.1158/1541-7786.MCR-20-0789 PMC8137548 33495399

[36] Miyazaki T Chung S Sakai H Ohata H Obata Y Shiokawa D. Stemness and immune evasion conferred by the TDO2-AHR pathway are associated with liver metastasis of colon cancer. Cancer Sci. (2022) 113:170–81. doi: 10.1111/cas.15182 PMC8748246 34714577

[37] Zhang X Liu X Zhou W Du Q Yang M Ding Y. Blockade of IDO-kynurenine-ahR axis ameliorated colitis-associated colon cancer via inhibiting immune tolerance. Cmgh. (2021) 12:1179–99. doi: 10.1016/j.jcmgh.2021.05.018 PMC8445903 34087454

[38] Ternes D Tsenkova M Pozdeev VI Meyers M Koncina E Atatri S. The gut microbial metabolite formate exacerbates colorectal cancer progression. Nat Metab. (2022) 4:458–75. doi: 10.1038/s42255-022-00558-0 PMC9046088 35437333

[39] Zhu P Yu H Zhou K Bai Y Qi R Zhang S. 3,3′-Diindolylmethane modulates aryl hydrocarbon receptor of esophageal squamous cell carcinoma to reverse epithelial-mesenchymal transition through repressing RhoA/ROCK1-mediated COX2/PGE2pathway. J Exp Clin Cancer Res. (2020) 39:1–18. doi: 10.1186/s13046-020-01618-7 32546278 PMC7298755

[40] Zhao Y Sun J Li Y Zhou X Zhai W Wu Y. Tryptophan 2,3-dioxygenase 2 controls M2 macrophages polarization to promote esophageal squamous cell carcinoma progression via AKT/GSK3β/IL-8 signaling pathway. Acta Pharm Sin B. (2021) 11:2835–49. doi: 10.1016/j.apsb.2021.03.009 PMC8463272 34589400

[41] Kuznetsov NV Andersson P Gradin K von Stein P Dieckmann A Pettersson S. The dioxin/aryl hydrocarbon receptor mediates downregulation of osteopontin gene expression in a mouse model of gastric tumourigenesis. Oncogene. (2005) 24:3216–22. doi: 10.1038/sj.onc.1208529 15735673

[42] Lai DW Liu SH Karlsson AI Lee WJ Wang KB Chen YC. The novel Aryl hydrocarbon receptor inhibitor biseugenol inhibits gastric tumor growth and peritoneal dissemination. Oncotarget. (2014) 5:7788–804. doi: 10.18632/oncotarget.2307 PMC4202161 25226618

[43] Wang K Li Y Jiang YZ Dai CF Patankar MS Song JS. An endogenous aryl hydrocarbon receptor ligand inhibits proliferation and migration of human ovarian cancer cells. Cancer Lett. (2013) 340:63–71. doi: 10.1016/j.canlet.2013.06.026 23851185 PMC3781955

[44] Litzenburger UM Opitz CA Sahm F Rauschenbach KJ Trump S Winter M. Constitutive IDO expression in human cancer is sustained by an autocrine signaling loop involving IL-6, STAT3 and the AHR. Oncotarget. (2014) 5:1038–51. doi: 10.18632/oncotarget.1637 PMC4011581 24657910

[45] Amobi-McCloud A Muthuswamy R Battaglia S Yu H Liu T Wang J. IDO1 expression in ovarian cancer induces PD-1 in T cells via aryl hydrocarbon receptor activation. Front Immunol. (2021) 12:678999. doi: 10.3389/fimmu.2021.678999 34025677 PMC8136272

[46] Son DS Roby KF Rozman KK Terranova PF. Estradiol enhances and estriol inhibits the expression of CYP1A1 induced by 2,3,7,8-tetrachlorodibenzo-p-dioxin in a mouse ovarian cancer cell line. Toxicology. (2002) 176:229–43. doi: 10.1016/S0300-483X(02)00162-2 12093619

[47] Li D Takao T Tsunematsu R Morokuma S Fukushima K Kobayashi H. Inhibition of AHR transcription by NF1C is affected by a single-nucleotide polymorphism, and is involved in suppression of human uterine endometrial cancer. Oncogene. (2013) 32:4950–9. doi: 10.1038/onc.2012.509 23208493

[48] Bian Y Li Y Shrestha G Wen X Cai B Wang K. ITE, an endogenous aryl hydrocarbon receptor ligand, suppresses endometrial cancer cell proliferation and migration. Toxicology. (2019) 421:1–8. doi: 10.1016/j.tox.2019.03.017 30953668

[49] DiNatale BC Smith K John K Krishnegowda G Amin SG Perdew GH. Ah receptor antagonism represses head and neck tumor cell aggressive phenotype. Mol Cancer Res. (2012) 10:1369–79. doi: 10.1158/1541-7786.MCR-12-0216 PMC3477495 22912337

[50] Frank DN Qiu Y Cao Y Zhang S Lu L Kofonow JM. A dysbiotic microbiome promotes head and neck squamous cell carcinoma. Oncogene. (2022) 41:1269–80. doi: 10.1038/s41388-021-02137-1 PMC8882136 35087236

[51] Stanford EA Ramirez-Cardenas A Wang Z Novikov O Alamoud K Koutrakis P. Role for the aryl hydrocarbon receptor and diverse ligands in oral squamous cell carcinoma migration and tumorigenesis. Mol Cancer Res. (2016) 14:696–706. doi: 10.1158/1541-7786.MCR-16-0069 27130942 PMC4987205

[52] Kenison JE Wang Z Yang K Snyder M Quintana FJ Sherr DH. The aryl hydrocarbon receptor suppresses immunity to oral squamous cell carcinoma through immune checkpoint regulation. Proc Natl Acad Sci U S A. (2021) 118. doi: 10.1073/pnas.2012692118 PMC8126867 33941684

[53] Wang LT Chiou SS Chai CY Hsi E Wang SN Huang SK. Aryl hydrocarbon receptor regulates histone deacetylase 8 expression to repress tumor suppressive activity in hepatocellular carcinoma. Oncotarget. (2017) 8:7489–501. doi: 10.18632/oncotarget.9841 PMC5352337 27283490

[54] Moennikes O Loeppen S Buchmann A Andersson P Ittrich C Poellinger L. A constitutively active dioxin/aryl hydrocarbon receptor promotes hepatocarcinogenesis in mice. Cancer Res. (2004) 64:4707–10. doi: 10.1158/0008-5472.CAN-03-0875 15256435

[55] Fan Y Boivin GP Knudsen ES Nebert DW Xia Y Puga A. The aryl hydrocarbon receptor functions as a tumor suppressor of liver carcinogenesis. Cancer Res. (2010) 70:212–20. doi: 10.1158/0008-5472.CAN-09-3090 PMC2939500 19996281

[56] Li L Wang T Li S Chen Z Wu J Cao W. TDO2 promotes the EMT of hepatocellular carcinoma through kyn-ahR pathway. Front Oncol. (2021) 10:562823. doi: 10.3389/fonc.2020.562823 33542896 PMC7851084

[57] Yu C Rao D Zhu H Liu Q Huang W Zhang L. TDO2 Was Downregulated in Hepatocellular Carcinoma and Inhibited Cell Proliferation by Upregulating the Expression of p21 and p27. BioMed Res Int. (2021) 2021:8–13. doi: 10.1155/2021/4708439 PMC8378971 34423034

[58] Chen W Wen L Bao Y Tang Z Zhao J Zhang X. Gut flora disequilibrium promotes the initiation of liver cancer by modulating tryptophan metabolism and up-regulating SREBP2. Proc Natl Acad Sci. (2022) 119. doi: 10.1073/pnas.2203894119 PMC9907126 36534812

[59] Koliopanos A Kleeff J Xiao Y Safe S Zimmermann A Büchler MW. Increased arylhydrocarbon receptor expression offers a potential therapeutic target for pancreatic cancer. Oncogene. (2002) 21:6059–70. doi: 10.1038/sj.onc.1205633 12203118

[60] Liang H Li T Fang X Zhang S Shi L Li W. IDO1/TDO dual inhibitor RY103 targets Kyn-AhR pathway and exhibits preclinical efficacy on pancreatic cancer. Cancer Lett. (2021) 522:32–43. doi: 10.1016/j.canlet.2021.09.012 34520819

[61] Wang L Tang W Yang S He P Wang J Gaedcke J. NO•/RUNX3/kynurenine metabolic signaling enhances disease aggressiveness in pancreatic cancer. Int J Cancer. (2020) 146:3160–9. doi: 10.1002/ijc.32733 PMC8189162 31609478

[62] Hezaveh K Shinde RS Klötgen A Halaby MJ Lamorte S Ciudad MT. Tryptophan-derived microbial metabolites activate the aryl hydrocarbon receptor in tumor-associated macrophages to suppress anti-tumor immunity. Immunity. (2022) 55:324–340.e8. doi: 10.1016/j.immuni.2022.01.006 35139353 PMC8888129

[63] Ye M Zhang Y Gao H Xu Y Jing P Wu J. Activation of the aryl hydrocarbon receptor leads to resistance to EGFR TKIs in non–small cell lung cancer by activating src-mediated bypass signaling. Clin Cancer Res. (2018) 24:1227–39. doi: 10.1158/1078-0432.CCR-17-0396 29229632

[64] Wang GZ Zhang L Zhao XC Gao SH Qu LW Yu H. The Aryl hydrocarbon receptor mediates tobacco-induced PD-L1 expression and is associated with response to immunotherapy. Nat Commun. (2019) 10:1–13. doi: 10.1038/s41467-019-08887-7 30850589 PMC6408580

[65] Nothdurft S Thumser-Henner C Breitenbücher F Okimoto RA Dorsch M Opitz CA. Functional screening identifies aryl hydrocarbon receptor as suppressor of lung cancer metastasis. Oncogenesis. (2020) 9:1–12. doi: 10.1038/s41389-020-00286-8 PMC7677369 33214553

[66] Nacarino-Palma A Rejano-Gordillo CM González-Rico FJ Ordiales-Talavero A Román ÁC Cuadrado M. Loss of aryl hydrocarbon receptor favors k-rasg12d-driven non-small cell lung cancer. Cancers (Basel). (2021) 13. doi: 10.3390/cancers13164071 PMC8394265 34439225

[67] Friedrich M Sankowski R Bunse L Kilian M Green E Ramallo Guevara C. Tryptophan metabolism drives dynamic immunosuppressive myeloid states in IDH-mutant gliomas. Nat Cancer. (2021) 2:723–40. doi: 10.1038/s43018-021-00201-z 35121943

[68] Takenaka MC Gabriely G Rothhammer V Mascanfroni ID Wheeler MA Chao CC. Control of tumor-associated macrophages and T cells in glioblastoma via AHR and CD39. Nat Neurosci. (2019) 22:1533. doi: 10.1038/s41593-019-0446-8 PMC9811951 31197266

[69] Jin UH Karki K Cheng Y Michelhaugh SK Mittal S Safe S. The aryl hydrocarbon receptor is a tumor suppressor-like gene in glioblastoma. J Biol Chem. (2019) 294:11342–53. doi: 10.1074/jbc.RA119.008882 PMC6643041 31171720

[70] Chaudhry KA Jacobi JJ Gillard BM Karasik E Martin JC da Silva Fernandes T. Aryl hydrocarbon receptor is a tumor promoter in MYCN-amplified neuroblastoma cells through suppression of differentiation. iScience. (2023) 26:108303. doi: 10.1016/j.isci.2023.108303 38026169 PMC10654598

[71] Morales-Hernández A Corrales-Redondo M Marcos-Merino JM González-Rico FJ Sánchez-Martín FJ Merino JM. AhR-dependent 2,3,7,8-tetrachlorodibenzo-p-dioxin toxicity in human neuronal cell line SHSY5Y. Neurotoxicology. (2016) 56:55–63. doi: 10.1016/j.neuro.2016.07.001 27392949

[72] Wu PY Liao YF Juan HF Huang HC Wang BJ Lu YL. Aryl hydrocarbon receptor downregulates MYCN expression and promotes cell differentiation of neuroblastoma. PloS One. (2014) 9:3–11. doi: 10.1371/journal.pone.0088795 PMC3931655 24586395

[73] Wu PY Yu IS Lin YC Chang YT Chen CC Lin KH. Activation of aryl hydrocarbon receptor by kynurenine impairs progression and metastasis of neuroblastoma. Cancer Res. (2019) 79:5550–62. doi: 10.1158/0008-5472.CAN-18-3272 31431462

[74] Fritz WA Lin TM Cardiff RD Peterson RE. The aryl hydrocarbon receptor inhibits prostate carcinogenesis in TRAMP mice. Carcinogenesis. (2007) 28:497–505. doi: 10.1093/carcin/bgl179 17052998

[75] Fritz WA Lin TM Safe S Moore RW Peterson RE. The selective aryl hydrocarbon receptor modulator 6-methyl-1,3,8-trichlorodibenzofuran inhibits prostate tumor metastasis in TRAMP mice. Biochem Pharmacol. (2009) 77:1151–60. doi: 10.1016/j.bcp.2008.12.015 PMC2659686 19166822

[76] Chen Z Cai A Zheng H Huang H Sun R Cui X. Carbidopa suppresses prostate cancer via aryl hydrocarbon receptor-mediated ubiquitination and degradation of androgen receptor. Oncogenesis. (2020) 9:1–13. doi: 10.1038/s41389-020-0236-x PMC7220950 32404918

[77] Richmond O Ghotbaddini M Allen C Walker A Zahir S Powell JB. The aryl hydrocarbon receptor is constitutively active in advanced prostate cancer cells. PloS One. (2014) 9. doi: 10.1371/journal.pone.0095058 PMC3995675 24755659

[78] Law MH Bishop DT Lee JE Brossard M Martin NG Moses EK. Genome-wide meta-analysis identifies five new susceptibility loci for cutaneous Malignant melanoma. Nat Genet. (2015) 47:987–95. doi: 10.1038/ng.3373 PMC4557485 26237428

[79] Corre S Tardif N Mouchet N Leclair HM Boussemart L Gautron A. Sustained activation of the Aryl hydrocarbon Receptor transcription factor promotes resistance to BRAF-inhibitors in melanoma. Nat Commun. (2018) 9:1–13. doi: 10.1038/s41467-018-06951-2 PMC6235830 30429474

[80] Contador-Troca M Alvarez-Barrientos A Barrasa E Rico-Leo EM Catalina-Fernández I Menacho-Márquez M. The dioxin receptor has tumor suppressor activity in melanoma growth and metastasis. Carcinogenesis. (2013) 34:2683–93. doi: 10.1093/carcin/bgt248 23843039

[81] Bender MJ McPherson AC Phelps CM Pandey SP Laughlin CR Shapira JH. Dietary tryptophan metabolite released by intratumoral Lactobacillus reuteri facilitates immune checkpoint inhibitor treatment. Cell. (2023) 186:1846–1862.e26. doi: 10.1016/j.cell.2023.03.011 37028428 PMC10148916

[82] Vlaar JM Borgman A Kalkhoven E Westland D Besselink N Shale C. Recurrent exon-deleting activating mutations in AHR act as drivers of urinary tract cancer. Sci Rep. (2022) 12:1–12. doi: 10.1038/s41598-022-14256-0 35710704 PMC9203531

[83] Shi MJ Meng XY Fontugne J Chen CL Radvanyi F Bernard-Pierrot I. Identification of new driver and passenger mutations within APOBEC-induced hotspot mutations in bladder cancer. Genome Med. (2020) 12:1–20. doi: 10.1186/s13073-020-00781-y PMC7646471 32988402

[84] Ishida M Mikami S Kikuchi E Kosaka T Miyajima A Nakagawa K. Activation of the aryl hydrocarbon receptor pathway enhances cancer cell invasion by upregulating the MMP expression and is associated with poor prognosis in upper urinary tract urothelial cancer. Carcinogenesis. (2010) 31:287–95. doi: 10.1093/carcin/bgp222 19755661

[85] Griffith BD Frankel TL. The aryl hydrocarbon receptor: impact on the tumor immune microenvironment and modulation as a potential therapy. Cancers (Basel). (2024) 16:1–19. doi: 10.3390/cancers16030472 PMC10854841 38339226

[86] Basson C Serem JC Hlophe YN Bipath P. The tryptophan–kynurenine pathway in immunomodulation and cancer metastasis. Cancer Med. (2023) 12:18691–701. doi: 10.1002/cam4.6484 PMC10557908 37644823

[87] Stone TW Williams RO. Modulation of T cells by tryptophan metabolites in the kynurenine pathway. Trends Pharmacol Sci. (2023) 44:442–56. doi: 10.1016/j.tips.2023.04.006 37248103

[88] Congues F Wang P Lee J Lin D Shahid A. Targeting aryl hydrocarbon receptor to prevent cancer in barrier organs. Biochem Pharmacol Published Online. (2024), 116156. doi: 10.1016/j.bcp.2024.116156 PMC11144369 38518996

[89] Martin JC da Silva Fernandes T Chaudhry KA Oshi M Abrams SI Takabe K. Aryl hydrocarbon receptor suppresses STING-mediated type I IFN expression in triple-negative breast cancer. Sci Rep. (2024) 14:1–13. doi: 10.1038/s41598-024-54732-3 38459088 PMC10923803

[90] Wu D Wang G Wen S Liu X He Q. ARID5A stabilizes Indoleamine 2,3-dioxygenase expression and enhances CAR T cell exhaustion in colorectal cancer. Transl Oncol. (2024) 42:101900. doi: 10.1016/j.tranon.2024.101900 38316094 PMC10862068

[91] Dumbrava EE Cecchini M Zugazagoitia J Lopez JS Jager D Olivia M. Initial results from a first-in-human, phase I study of immunomodulatory aryl hydrocarbon receptor (AhR) inhibitor BAY2416964 in patients with advanced solid tumors. J Clin Oncol. (2023) 41:2502–2. doi: 10.1200/jco.2023.41.16_suppl.2502

[92] Shin JH Zhang L Murillo-Sauca O Kim J Kohrt HEK Bui JD. Modulation of natural killer cell antitumor activity by the aryl hydrocarbon receptor. Proc Natl Acad Sci U S A. (2013) 110:12391–6. doi: 10.1073/pnas.1302856110 PMC3725066 23836658

[93] Sato K Ohira M Imaoka Y Imaoka K Bekki T Doskali M. The aryl hydrocarbon receptor maintains antitumor activity of liver resident natural killer cells after partial hepatectomy in C57BL/6J mice. Cancer Med. (2023) 12:19821–37. doi: 10.1002/cam4.6554 PMC10587932 37747052

[94] Trikha P Moseman JE Thakkar A Campbell AR Elmas E Foltz JA. Defining the AHR-regulated transcriptome in NK cells reveals gene expression programs relevant to development and function. Blood Adv. (2021) 5:4605–18. doi: 10.1182/bloodadvances.2021004533 PMC8759121 34559190

[95] Boitano AE. Aryl hydrocarbon receptor antagonists promote the expansion of human hematopoietic stem cells (Science (2010) (1345)). Sci (80-). (2011) 332:664. doi: 10.1126/science.1191536 PMC3033342 20688981

[96] Kober C Roewe J Schmees N Roese L Roehn U Bader B. Targeting the aryl hydrocarbon receptor (AhR) with BAY 2416964: a selective small molecule inhibitor for cancer immunotherapy. J Immunother Cancer. (2023) 11:e007495. doi: 10.1136/jitc-2023-007495 37963637 PMC10649913

[97] McGovern K Castro AC Cavanaugh J Coma S Walsh M Tchaicha J. Discovery and characterization of a novel aryl hydrocarbon receptor inhibitor, IK-175, and its inhibitory activity on tumor immune suppression. Mol Cancer Ther. (2022) 4:1261–72. doi: 10.1158/1535-7163.MCT-21-0984 35666806

[98] Lebwohl MG Stein Gold L Strober B Papp KA Armstrong AW Bagel J. Phase 3 trials of tapinarof cream for plaque psoriasis. N Engl J Med. (2021) 385:2219–29. doi: 10.1056/nejmoa2103629 34879448

[99] Chen Z Xia X Chen H Huang H An X Sun M. Carbidopa suppresses estrogen receptor-positive breast cancer via AhR-mediated proteasomal degradation of ERα. Invest New Drugs. (2022) 40:1216–30. doi: 10.1007/s10637-022-01289-5 36070108

[100] Ogura J Miyauchi S Shimono K Yang S Gonchigar S Ganapathy V. Carbidopa is an activator of aryl hydrocarbon receptor with potential for cancer therapy. Biochem J. (2017) 474:3391–402. doi: 10.1042/BCJ20170583 28963435

[101] Park H Jin UH Karki K Jayaraman A Allred C Michelhaugh SM. Dopamine is an aryl hydrocarbon receptor agonist. Biochem J. (2020) 477:3899–910. doi: 10.1042/BCJ20200440 PMC7772691 32905582

[102] Koch DC Jang HS O’Donnell EF Punj S Kopparapu PR Bisson WH. Anti-androgen flutamide suppresses hepatocellular carcinoma cell proliferation via the aryl hydrocarbon receptor mediated induction of transforming growth factor-β1. Oncogene. (2015) 34:6092–104. doi: 10.1038/onc.2015.55 25867062

[103] O’Donnell EF Kopparapu PR Koch DC Jang HS Phillips JL Tanguay. The Aryl hydrocarbon receptor mediates leflunomide-induced growth inhibition of melanoma cells. PloS One. (2012) 7. doi: 10.1371/journal.pone.0040926 PMC3398955 22815870

[104] Jin UH Lee SO Safe S. Aryl hydrocarbon receptor (AHR)-active pharmaceuticals are selective AHR modulators in MDA-MB-468 and BT474 breast cancer cells. J Pharmacol Exp Ther. (2012) 343:333–41. doi: 10.1124/jpet.112.195339 PMC3477220 22879383

[105] Jin UH Michelhaugh SK Polin LA Shrestha R Mittal S Safe S. Omeprazole inhibits glioblastoma cell invasion and tumor growth. Cancers (Basel). (2020) 12:1–15. doi: 10.3390/cancers12082097 PMC7465678 32731514

[106] Bai Y Zhu P Zhou K Zhang S-G. Effect of the acid suppressor omeprazole on the proliferation, migration, invasion and cell cycle of esophageal squamous cell carcinoma cells via the aryl hydrocarbon receptor pathway. Exp Ther Med. (2021) 22. doi: 10.3892/etm.2021.10621 PMC8406681 34475977

[107] Jin UH Kim SB Safe S. Omeprazole inhibits pancreatic cancer cell invasion through a nongenomic aryl hydrocarbon receptor pathway. Chem Res Toxicol. (2015) 28:907–18. doi: 10.1021/tx5005198 PMC4948974 25826687

[108] O’Donnell EF Koch DC Bisson WH Jang HS Kolluri SK. The aryl hydrocarbon receptor mediates raloxifene-induced apoptosis in estrogen receptor-negative hepatoma and breast cancer cells. Cell Death Dis. (2014) 5:1–12. doi: 10.1038/cddis.2013.549 PMC4040680 24481452

[109] Wei KL Gao GL Chou YT Lin CY Chen SC Chen YL. Sorafenib is an antagonist of the aryl hydrocarbon receptor. Toxicology. (2022) +):153118. doi: 10.1016/j.tox.2022.153118 35124147

[110] Ciolino HP Bass SE MacDonald CJ Cheng RYS Yeh GC. Sulindac and its metabolites induce carcinogen metabolizing enzymes in human colon cancer cells. Int J Cancer. (2008) 122:990–8. doi: 10.1002/ijc.23218 17985343

